# Evidence on the accreditation of health professionals’ education in the World Health Organization Africa region: a scoping review protocol

**DOI:** 10.11124/JBIES-24-00285

**Published:** 2025-04-07

**Authors:** Catherine Nakidde, Debora Marletta, Gerry McGivern, Catherine O’Keeffe, Ann Griffin

**Affiliations:** 1 Research Department of Medical Education, UCL Medical School, University College London, London, UK; 2 UCL LCCOS - Library, Culture, Collections and Open Science, University College London, London, UK; 3 King’s Business School, Kings College London, London, UK

**Keywords:** accreditation, Africa, health professionals’ education, quality assurance

## Abstract

**Objective:**

This scoping review aims to map and examine the extent and type of available evidence on health professionals’ education accreditation within Africa.

**Introduction:**

The demand for health professionals is unprecedentedly high globally. One response to this challenge has been expanding training through more liberal education policies, facilitating private sector participation in education service provision. Some evidence suggests that this is a double-edged sword, increasing quantity but compromising the quality of health professionals produced. Regulation can provide a framework to assure and continuously improve quality, with such regulation in place in 79% of World Health Organization African countries. However, it is unclear how much and what evidence has been generated on how accreditation happens, where it is concentrated, and the prevailing evidence gaps within this region; therefore, we propose to conduct a scoping review.

**Inclusion criteria:**

This review will include articles and dissertations focusing on the accreditation of health professionals’ education in Africa. All methodological approaches and designs will be included. Conference abstracts and protocols will be excluded.

**Methods:**

This review will be carried out according to the JBI scoping review methodology. We conducted an initial search of CINAHL and MEDLINE to identify relevant articles. This informed our selection of keywords, along with index terms, to create a comprehensive search strategy for CINAHL (EBSCOhost), MEDLINE (Ovid), Global Health (Ovid), ERIC (EBSCOhost), Web of Science Core Collection, Embase, and Scopus. Sources included will be limited to those published from 2000 onwards. Data will be presented using tables and charts, accompanied by a narrative summary.

**Review registration:**

Open Science Framework https://osf.io/w5g7t

## Introduction

The inadequate number of key health workers globally is a crisis that spans decades. Within the World Health Organization (WHO) African region, the density of doctors, nurses, and midwives is 1.55 per 1000 population, with the region projected to have 52% of the global shortage of health workers by 2030.^1^,^2^ Without the appropriate numbers, skill mix, and efficient distribution of health workers, health systems cannot function optimally.^3^,^4^ Countries have attempted to solve this problem with a blend of solutions, with the most prevalent one being increasing the number of health workers trained through education reforms or liberalization policies, including the commercialization and privatization of education (neoliberalism).^5^,^6^

Neoliberalism is manifested in policies that favor privatization, deregulation of industry, tax cuts, and competition and profit-making in sectors such as education and health care that are considered public services.^7^ Hogan and Thompson define the commercialization of education as the “creation, marketing and sale of education goods and services for commercial gain.”^8^^(p.5)^ Commercialization of higher education is documented across the world but more prominently in the global south—70% of private universities are within the global south.^6^,^9^–^11^ However, contrary to its intended purpose, liberalization is reported to have exacerbated inequity and exclusion, lowered quality, and maligned higher education and the developmental priorities for developing countries.^12^,^13^ In sub-Saharan Africa, particularly, most jurisdictions are implementing these policies but with limited capacity and resources to safeguard the quality of education compared with the developed countries.^13^,^14^

In contrast, when it comes to health professionals’ education (HPE), privately owned training institutions have played an important role in increasing the number of health care professionals in many countries.^9^,^10^,^15^,^16^ However, the quantitative increase in health workers has, on many occasions, not been matched with equal efforts to ensure the quality of graduates that are produced.^16^,^17^ In many low- and middle-income countries (LMICs), 15% of mortality is attributed to low-quality care, which is the result not only of systemic constraints, such as lack of critical equipment and medicines, but, crucially, an inadequately trained workforce contributing to low quality of care.^18^,^19^ In the WHO Africa region, low-quality care exacerbates the high burden of disease, further straining health systems and contributing to familial poverty through lost productivity and long-term disability.^1^,^18^

Regulation of HPE through accreditation can provide a framework to assure and continuously improve quality within education. In this protocol, HPE refers to medical, pharmacy, nursing, midwife, physiotherapy, dental, nutrition, occupational therapy, and allied health professions (health associate professions) education. Accreditation refers to “the process of formal evaluation of an educational program, institution, or system against defined standards by an external body for the purposes of quality assurance and continuous enhancement.”^20^ ^(p.4)^ How this process unfolds usually depends on the jurisdiction in question; however, there are key vital elements that are common across HPE systems. These include i) accreditation standards or benchmarks used to judge the quality of a program or institution; ii) a self-study or evaluation, which is an inhouse process conducted by the program or institution to assess their compliance with the accreditation standards; iii) a peer review, which is an external evaluation of the program or institution conducted by a team commissioned by the accrediting body, resulting in an accreditation report; and iv) an accreditation decision made by the accrediting body.^20^

However, many LMICs continue to grapple with formidable contextual issues such as insufficient monetary and human resources within regulatory agencies and corruption, which weakens implementation.^21^,^22^ Some evidence suggests that there are key challenges with quality assurance and the regulation of private universities and training colleges that provide HPE in Africa. This indicates the need for further research on HPE accreditation in this region.^3^,^16^,^22^,^23^ While Okoroafor and colleagues^24^ report that 79% of the countries within the WHO African region have HPE accreditation mechanisms in place, it is unclear how much research has been conducted to document and examine how the regulation of HPE occurs, where this evidence is concentrated within the region, and the prevailing gaps. This is why we are proposing to conduct a scoping review, which is used in instances where there is a need to examine the scope and type of evidence on a given topic and identify knowledge gaps.^25^

Most of the indexed evidence on the accreditation of HPE comes from Western countries and there are notable evidence gaps in LMICs that need attention. Within LMICs, the WHO African region is still underrepresented; however, the extent of this gap can only be determined by using systematic methods that a scoping review applies. Therefore, the aim of this review is to map and examine the extent and type of available evidence on the accreditation of HPE within the WHO African region. The review will highlight evidence gaps, such as which accreditation elements are least studied, where evidence is least produced, and the types of studies that need to be conducted. These gaps will inform future research on the regulation of HPE in the region and ultimately contribute to strengthening HPE regulation.

A preliminary search of MEDLINE, CINAHL, and *JBI Evidence Synthesis* was conducted and no current or in-progress systematic or scoping reviews on the topic were identified. This review forms part of a larger PhD project where the researcher focuses on the accreditation of HPE in one African country. Therefore, reviewing the evidence within Africa will provide a wider contextual background for the PhD project, with the review findings shaping the trajectory of the rest of the study.

## Review questions

- How much and what type of evidence is available on HPE accreditation in Africa?
- In which African countries is this evidence concentrated?
- What are the existing gaps in evidence?

## Inclusion criteria

### Participants

This review will focus on studies that include regulators (individuals who implement HPE accreditation), HPE service providers (health training institutions or their representatives), and HPE students in any country within the WHO African region. For this review, HPE includes medical, pharmacy, nursing, midwife, physiotherapy, dental, nutrition, occupational therapy, and allied health professions (health associate professions) education.

### Concept

This review will consider research that examines the elements involved (such as minimum standards) and processes (such as self-assessments, peer reviews, site visits, accreditation cycles) that are carried out to improve and assure the quality of education programs, institutions, and systems in which health professionals study before entering their professions. Core accreditation elements defined by the International Health Professions Accreditation Outcomes Consortium and documented by Frank and colleagues^20^ will guide which evidence on accreditation to include in this review. A list and definitions of these elements can be found at https://bmcmededuc.biomedcentral.com/articles/10.1186/s12909-020-02121-5/tables/4.

### Context

The geographical context for this study is the WHO African region and, therefore, evidence from these African countries will be eligible for inclusion if it meets all the other inclusion criteria.

### Types of sources

The review will consider evidence that is quantitative, qualitative, or mixed methods and produced using research designs including, but not limited to, experimental studies, analytical observation studies, analytical cross-sectional studies, case studies, ethnography, grounded theory, and phenomenology. Additionally, any form of review that meets the eligibility criteria will be considered. We shall include all types of articles, including descriptive, theoretical, and empirical research studies. The sources of information will include scholarly journal articles (which could be primary research studies or reviews), research reports, theses, and dissertations. Protocols and conference abstracts will be excluded.

## Methods

The proposed review will be carried out according to the JBI scoping review methodology authored by Peters and colleagues^26^ and will be reported according to the Preferred Reporting Items for Systematic Reviews and Meta-Analyses extension for Scoping Reviews (PRISMA-ScR).^27^

### Search strategy

A 3-phase search strategy will be employed to locate both published and unpublished papers. An initial limited search of CINAHL Plus (EBSCOhost) and MEDLINE (Ovid) was undertaken to identify articles related to the topic. In the second phase, the keywords contained in the titles and abstracts of the relevant articles, and the index terms used to describe them, were used to develop a full search strategy for the different databases to be searched (see Appendix I). This search strategy, including all identified keywords and index terms, will be adapted for each included database and/or information source. In the third phase, the reference lists of all included sources of evidence will be screened for additional studies. The reviewers also intend to consult authors of primary studies or reviews for further information, where relevant. This process has ongoing support from a librarian.

All research published from the year 2000 to date will be included, as evidence shows that most accreditation systems in LMICs were established between 2000 and 2010.^28^ For research that is not published in English, we will use online translation tools or contact authors for English versions of the publications. The only gray literature that will be included are evaluation/research reports, dissertations, and theses.

The following databases will be searched: CINAHL Plus (EBSCOhost), MEDLINE (Ovid), Global Health (Ovid), Eric (EBSCOhost), Web of Science Core Collection, Embase, and Scopus. The following sources of gray literature will be searched: the World Bank website, the WHO website, Overton, Policy Commons, and ProQuest Dissertations and Theses.

### Study selection

After the search, all identified citations will be imported and uploaded into EndNote v.21 (Clarivate Analytics, PA, USA) and duplicates will be removed. A pilot test will then be conducted, which will involve screening 10% of titles and abstracts against the inclusion criteria by 2 independent reviewers. This will be followed by discussion of the results and modification of the inclusion criteria and the elaboration document with the rest of the reviewers.

Following this pilot test, titles and abstracts of all included sources will be exported to Rayyan (Qatar Computing Research Institute, Doha, Qatar) for assessment against the inclusion criteria by 2 reviewers. The full text of selected citations will be uploaded into Rayyan and assessed in detail against the inclusion criteria by 2 independent reviewers. Reasons for the exclusion of sources of evidence at full text that do not meet the inclusion criteria will be recorded and reported in the scoping review. Any disagreements that arise between the reviewers at each stage of the selection process will be resolved through discussion or with an additional reviewer. The results of the search and the study inclusion process will be reported in full in the final scoping review and presented in a PRISMA flow diagram.^29^ We will not appraise the methodological quality or risk of bias for the studies included in our review, which is in line with scoping review guidelines.^27^

### Data extraction

Data will be extracted using a modified JBI data charting table (see Appendix II). This will be piloted by 2 reviewers using 5 studies to ensure consistency, and may be iteratively modified as the review progresses. The data extraction form will be used to record all essential information about each source of information (participants, concept, context, study methods, etc.). We will also extract data based on Frank and others’^20^ essential elements of accreditation systems: i) accreditation mandate, ii) standards, iii) application for accreditation, iv) self-study or self-evaluation, v) peer review or external assessment of compliance with standards, vi) accreditation reports, vii) accreditation decisions, viii) accreditation cycle, ix) site review model, x) accreditation system administration, and other findings relevant to the research questions. Data will be extracted by 2 independent reviewers using this data extraction tool and managed using Microsoft Excel (Redmond, Washington, USA). In cases of missing data, a reviewer will contact the authors up to 3 times by email. And disagreements between reviewers will be resolved through discussion or with a third reviewer if necessary.

### Data analysis and presentation

After all the data have been collected, the reviewers will decide on the presentation and analysis of the data. We anticipate conducting basic descriptive analysis for all subquestions and basic content analysis for part of subquestion 1 in which we will categorize evidence based on the elements of accreditation studied within the different countries. To present our findings, we will use summative descriptive statistics figures and tables for accreditation elements that have been studied as well as characteristics of included articles and concept maps to present key information about the sources of information (eg, geographical context, methodologies applied, participants). These will be accompanied by narrative summaries to describe the relation between the review questions and the results.

## Acknowledgments

The proposed review is part of a doctoral project and will contribute to a doctoral degree for CN.

## Funding

The Commonwealth Scholarship Commission has provided funding for a doctoral degree for CN.

## Author contributions

CN designed the study and developed the first draft of the protocol, with critical input from AG, GM, and CO. CN and DM developed and conducted the search strategy. All authors have read and approved the final manuscript.

## Appendix I: Search strategy

### CINAHL Plus (EBSCOhost)

Date searched: July 21, 2024

**Table:**

Search	Query	Records retrieved
#1	TI accredit* OR AB accredit*	12,968
#2	TI regulat* OR AB regulat*	171,450
#3	TI quality assurance OR AB quality assurance	6282
#4	(MH “Accreditation+“)	19,737
#5	S1 OR S2 OR S3 OR S4	203,770
#6	TI health profession* OR AB health profession*	78,763
#7	TI medical OR AB medical	420,986
#8	TI pharmac* OR AB pharmac*	154,973
#9	TI nurs* OR AB nurs*	459,392
#10	TI midwife* OR AB midwife*	18,616
#11	TI physiotherap* OR AB physiotherap*	24,355
#12	TI dent* OR AB dent*	92,947
#13	TI nutrition* OR AB nutrition*	118,213
#14	TI occupational therap* OR AB occupational therap*	23,040
#15	TI allied health profession* OR AB allied health profession*	2777
#16	TI health associate profession* OR AB health associate profession*	30
#17	(MH “Allied Health Professions+“) OR (MH “Health Occupations+“)	962,977
#18	S6 OR S7 OR S8 OR S9 OR S10 OR S11 OR S12 OR S13 OR S14 OR S15 OR S16 OR S17	1,961,306
#19	TI education OR AB education	297,372
#20	TI training OR AB training	223,186
#21	(MH “Education+“)	1,068,560
#22	S19 OR S20 OR S21	1,321,826
#23	(Africa*) or ((ZZ “africa”))	113,059
#24	(MH “Africa+“) OR (MH “Algeria”) OR (MH “Cameroon”) OR (MH “Central African Republic”) OR (MH “Chad”) OR (MH “Congo”) OR (MH “Democratic Republic of the Congo”) OR (MH “Equatorial Guinea”) OR (MH “Gabon”) OR (MH “Burundi”) OR (MH “Eritrea”) OR (MH “Ethiopia”) OR (MH “Kenya”) OR (MH “Rwanda”) OR (MH “Tanzania”) OR (MH “Uganda”) OR (MH “Angola”) OR (MH “Botswana”) OR (MH “Lesotho”) OR (MH “Malawi”) OR (MH “Mozambique”) OR (MH “Namibia”) OR (MH “South Africa”) OR (MH “Swaziland”) OR (MH “Zambia”) OR (MH “Zimbabwe”) OR (MH “Benin”) OR (MH “Burkina Faso”) OR (MH “Cape Verde”) OR (MH “Cote d’Ivoire”) OR (MH “Gambia”) OR (MH “Ghana”) OR (MH “Guinea”) OR (MH “Guinea-Bissau”) OR (MH “Liberia”) OR (MH “Mali”) OR (MH “Mauritania”) OR (MH “Niger”) OR (MH “Nigeria”) OR (MH “Senegal”) OR (MH “Sierra Leone”) OR (MH “Togo”)	97,881
#25	TI (Africa* or Algeria or Angola or Benin or Botswana or Burundi or Cabo Verde or Cameroon or “Central African Republic” or Chad or Comoros or “Democratic Republic of Congo” or “Republic of the Congo” or Cote d’Ivoire or Equatorial Guinea or Eritrea or Eswatini or Ethiopia or Gabon or Gambia or Ghana or Guinea or Guinea-Bissau or Kenya or Lesotho or Liberia or Madagascar or Malawi or Mali or Mauritania or Mauritius or Mozambique or Namibia or Niger or Nigeria or Rwanda or “Sao Tome and Principe” or Senegal or Seychelles or Sierra Leone or South Africa or “South Sudan” or Tanzania or Togo or Uganda or Zambia or Zimbabwe) OR AB (Africa or Algeria or Angola or Benin or Botswana or Burundi or Cabo Verde or Cameroon or “Central African Republic” or Chad or Comoros or “Democratic Republic of Congo” or “Republic of the Congo” or Cote d’Ivoire or Equatorial Guinea or Eritrea or Eswatini or Ethiopia or Gabon or Gambia or Ghana or Guinea or Guinea-Bissau or Kenya or Lesotho or Liberia or Madagascar or Malawi or Mali or Mauritania or Mauritius or Mozambique or Namibia or Niger or Nigeria or Rwanda or “Sao Tome and Principe” or Senegal or Seychelles or Sierra Leone or South Africa or “South Sudan” or Tanzania or Togo or Uganda or Zambia or Zimbabwe)	132,388
#26	S23 OR S24 OR S25	177,386
#27	S5 AND S18 AND S22 AND S26	566
Limited to publications starting the year 2000.		

## Appendix II: Draft data extraction instrument

**Scoping review details**

Scoping review title:

Review objective/s:

Review question/s:

**Inclusion/exclusion criteria**

Population

Concept

Context

Type of evidence source

**Evidence source details and characteristics**

Citation details (eg, author/s, date, title, journal, volume, issue, pages)

Country

Context

Participants details (eg, age/sex and number)

Methods used

**Details/results extracted from source of evidence (accreditation elements studied)**

1. Accreditation mandate
2. Standards
3. Application for accreditation
4. Self-study or self-evaluation
5. Peer-review or external assessment of compliance to standards
6. Accreditation reports
7. Accreditation decisions
8. Accreditation cycle
9. Site review model
10. Accreditation system administration

## References

[1] Ahmat A Okoroafor SC Kazanga I Asamani JA Millogo JJS Illou MMA . The health workforce status in the WHO African Region: findings of a cross-sectional study. BMJ Glob Health 2022;7(Suppl 1):e008317. 10.1136/bmjgh-2021-008317 PMC9109011 35675966

[2] Boniol M Kunjumen T Nair TS Siyam A Campbell J Diallo K . The global health workforce stock and distribution in 2020 and 2030: a threat to equity and ‘universal’ health coverage? BMJ Glob Health 2022;7(6):e009316. 10.1136/bmjgh-2022-009316 PMC9237893 35760437

[3] Frenk J Chen L Bhutta ZA Cohen J Crisp N Evans T . Health professionals for a new century: transforming education to strengthen health systems in an interdependent world. Lancet 2010;376(9756):1923–58. 21112623 10.1016/S0140-6736(10)61854-5

[4] World Health Organization . Global strategy on human resources for health: workforce 2030 [internet]. WHO; 2016 [cited 2024 Apr 9]. Available from: https://iris.who.int/bitstream/handle/10665/250368/9789241511131-eng.pdf.

[5] Frenk J Chen LC Chandran L Groff EOH King R Meleis A . Challenges and opportunities for educating health professionals after the COVID-19 pandemic. Lancet 2022;400(10362):1539–56. 36522209 10.1016/S0140-6736(22)02092-X PMC9612849

[6] Shehnaz SI . Privatisation of medical education: viewpoints with a global perspective. Sultan Qaboos Univ Med J 2010;10(1):6–11. 21509076 PMC3074658

[7] Peck J . Explaining (with) neoliberalism. Territ Polit Gov 2013;1(2):132–57.

[8] Hogan A Thompson G . Privatisation and commercialisation in public education: how the public nature of schooling is changing. Routledge; 2020.

[9] Mwita N Ngwawe C Okaro S The role of private sector training institutions in addressing nurse shortages in Kenya. Amref Health Africa; 2016 [cited 2025 Apr 5]. Available from: https://assets.publishing.service.gov.uk/media/57a0895d40f0b6497400003c/AMREF_HWbrief_4April.pdf.

[10] Sirili N Frumence G Kiwara A Mwangu M Goicolea I Hurtig A-K . Public private partnership in the training of doctors after the 1990s’ health sector reforms: the case of Tanzania. Hum Res Health 2019;17(33):1–11. 10.1186/s12960-019-0372-6 PMC6532226 31118038

[11] Bothwell E One in three students globally now in private higher education [internet]. Times Higher Education; 2018 [cited 2024 Feb 25]. Available from: https://www.timeshighereducation.com/news/one-three-students-globally-now-private-higher-education.

[12] Choudhury B . Public services and international trade liberalization: human rights and gender implications. Cambridge University Press; 2012.

[13] Ervjola S . Universities between the state and the market. SAIH; 2018.

[14] Masanja V Lwakabamba S , editors. Liberalization of higher education in Sub-Saharan Africa. RUFORUM Biennial Regional Conference, 17-21 October 2016, Cape Town, South Africa; 2016.

[15] Masselink LE Lee S-YD . Nurses, Inc.: expansion and commercialization of nursing education in the Philippines. Soc Sci Med 2010;71(1):166–72. 20399550 10.1016/j.socscimed.2009.11.043

[16] Reynolds J Wisaijohn T Pudpong N Watthayu N Dalliston A Suphanchaimat R . A literature review: the role of the private sector in the production of nurses in India, Kenya, South Africa and Thailand. Hum Res Health 2013;11(1):14. 10.1186/1478-4491-11-14 PMC3637479 23587128

[17] Aftab W Khan M Rego S Chavan N Rahman-Shepherd A Sharma I . Variations in regulations to control standards for training and licensing of physicians: a multi-country comparison. Hum Res Health 2021;19(1). 10.1186/s12960-021-00629-5 PMC8299694 34301245

[18] National Academies of Sciences Engineering and Medicine . Crossing the global quality chasm: improving health care worldwide. National Academies Press; 2018. 30605296

[19] World Health Organization . Quality health services [internet]. WHO; 2020 cited [2024 Jan 12]. Available from: https://www.who.int/news-room/fact-sheets/detail/quality-health-services

[20] Frank JR Taber S van Zanten M Scheele F Blouin D . The role of accreditation in 21st century health professions education: report of an International Consensus Group. BMC Med Ed 2020;20(305):1–9. 10.1186/s12909-020-02121-5 PMC7520947 32981519

[21] Sheikh K Saligram PS Hort K . What explains regulatory failure? Analysing the architecture of health care regulation in two Indian states. Health Pol Plan 2015;30(1):39–55. 10.1093/heapol/czt095 24342742

[22] McGivern G Wafula F Seruwagi G Kiefer T Musiega A Nakidde C . Deconcentrating regulation in low- and middle-income country health systems: a proposed ambidextrous solution to problems with professional regulation for doctors and nurses in Kenya and Uganda. Hum Res Health 2024;22(13):1–13. 10.1186/s12960-024-00891-3 PMC10835984 38308369

[23] Zhao Y Osano B Were F Kiarie H Nicodemo C Gathara D . Characterising Kenyan hospitals’ suitability for medical officer internship training: a secondary data analysis of a cross-sectional study. BMJ Open 2022;12(e056426):1–9. 10.1136/bmjopen-2021-056426 PMC9083393 35523483

[24] Okoroafor SC Ahmat A Asamani JA Millogo JJS Nyoni J . An overview of health workforce education and accreditation in Africa: implications for scaling-up capacity and quality. Hum Res Health 2022;20(37):1–7. 10.1186/s12960-022-00735-y PMC9077809 35525955

[25] Munn Z Peters MDJ Stern C Tufanaru C McArthur A Aromataris E . Systematic review or scoping review? Guidance for authors when choosing between a systematic or scoping review approach. BMC Med Res Methodol 2018;18(143):1–7. 30453902 10.1186/s12874-018-0611-x PMC6245623

[26] Peters MDJ Marnie C Tricco AC Pollock D Munn Z Alexander L . Updated methodological guidance for the conduct of scoping reviews. JBI Evid Synth 2020;18(10):2119–26. 33038124 10.11124/JBIES-20-00167

[27] Tricco AC Lillie E Zarin W O’Brien KK Colquhoun H Levac D . PRISMA extension for Scoping Reviews (PRISMA-ScR): checklist and explanation. Ann Intern Med 2018;169(7):467–73. 30178033 10.7326/M18-0850

[28] Bedoll D van Zanten M McKinley D . Global trends in medical education accreditation. Hum Res Health 2021;19(70):1–15. 10.1186/s12960-021-00588-x PMC8136216 34016122

[29] Page MJ McKenzie JE Bossuyt PM Boutron I Hoffmann TC Mulrow CD . The PRISMA 2020 statement: an updated guideline for reporting systematic reviews. BMJ 2021;372(71):1–9. 10.1136/bmj.n71 PMC8005924 33782057

